# Hamster and Murine Models of Severe Destructive Lyme Arthritis

**DOI:** 10.1155/2012/504215

**Published:** 2012-02-22

**Authors:** Erik Munson, Dean T. Nardelli, Brian K. Du Chateau, Steven M. Callister, Ronald F. Schell

**Affiliations:** ^1^Wheaton Franciscan Laboratory, 11020 West Plank Court, Suite 100, Wauwatosa, WI 53226, USA; ^2^College of Health Sciences, University of Wisconsin-Milwaukee, Milwaukee, WI 53201, USA; ^3^Dako North America, Inc., Carpinteria, CA 93013, USA; ^4^Section of Infectious Diseases, Gundersen Lutheran Medical Center, La Crosse, WI 54601, USA; ^5^Wisconsin State Laboratory of Hygiene, Madison, WI 53706, USA; ^6^Department of Microbiology, University of Wisconsin-Madison, Madison, WI 53706, USA; ^7^Department of Medical Microbiology and Immunology, University of Wisconsin-Madison, Madison, WI 53706, USA

## Abstract

Arthritis is a frequent complication of infection in humans with *Borrelia burgdorferi*. Weeks to months following the onset of Lyme borreliosis, a histopathological reaction characteristic of synovitis including bone, joint, muscle, or tendon pain may occur. A subpopulation of patients may progress to a chronic, debilitating arthritis months to years after infection which has been classified as severe destructive Lyme arthritis. This arthritis involves focal bone erosion and destruction of articular cartilage. Hamsters and mice are animal models that have been utilized to study articular manifestations of Lyme borreliosis. Infection of immunocompetent LSH hamsters or C3H mice results in a transient synovitis. However, severe destructive Lyme arthritis can be induced by infecting irradiated hamsters or mice and immunocompetent *Borrelia*-vaccinated hamsters, mice, and interferon-gamma- (IFN-*γ*-) deficient mice with viable *B. burgdorferi*. The hamster model of severe destructive Lyme arthritis facilitates easy assessment of Lyme borreliosis vaccine preparations for deleterious effects while murine models of severe destructive Lyme arthritis allow for investigation of mechanisms of immunopathology.

## 1. Introduction

### 1.1. Lyme Borreliosis Background

Since its discovery in the mid 1970s, Lyme borreliosis has become the most prevalent tick-borne infectious illness in the United States [1]. Following a brief early infection period that can be characterized by constitutional symptoms [2] and the pathognomonic erythema migrans [3], successive stages of the illness in nontreated individuals can consist of a dissemination of the etiologic agent *Borrelia burgdorferi* to multiple organ sites and subsequent persistence of the spirochete. Consequences of this activity are immunopathologies, with afflicted individuals presenting with cardiac [4], neurological [5, 6], and musculoskeletal [7] abnormalities.

### 1.2. Synovitis and Severe Destructive Arthritis in Humans

In early Lyme borreliosis, afflicted individuals may experience joint, tendon, muscle, or bone pain. Subsequently, fifty to sixty percent display acute signs of arthritis, including musculoskeletal pain without objective findings [7, 8]. Histopathological analyses of synovectomies and synovial biopsies at this stage of Lyme borreliosis articular involvement have described a hyperplastic synovitis [9]. Arthritic involvement in late Lyme disease includes intermittent episodes of joint pain, with 50–60% of such individuals suffering from inflammatory arthritis with synovial effusion or pain on motion [7, 8]. Of these patients, 11% may progress to a chronic and debilitating severe destructive Lyme arthritis [7]. The increased severity of arthropathy at this stage has been characterized as a synovial hyperplasia, an infiltration of mononuclear cells into the synovium, a formation of an inflammatory exudate over the lining of synovial cells (pannus formation), a destruction of cartilage, and as an erosion of bones and joints [10, 11].

Early associations were hypothesized between the major histocompatibility complex (MHC) class II alleles HLA-DR2 and HLA-DR4 and severe destructive Lyme arthritis [12, 13]. Furthermore, individuals afflicted with a severe destructive Lyme arthritis that was refractory to antimicrobial therapy predominately showed an HLA-DR4 specificity [14]. Gross et al. [15] subsequently identified an immunodominant epitope within *B. burgdorferi* outer surface protein A (OspA) for T lymphocytes that may shoulder responsibility for the induction of treatment-resistant severe destructive Lyme arthritis. In support of this statement, Steere et al. [16] showed that the majority of patients with late-stage, antimicrobial-refractory Lyme arthritis possessed at least one of the HLA-DR molecules known to bind to this putative arthritogenic epitope of OspA while those individuals with antimicrobial-responsive arthritis possessed these molecules to a significantly less-frequent degree. Moreover, extensive investigations into identifying additional arthritogenic, T lymphocyte-binding epitopes of *B. burgdorferi* [17] yielded no more suitable candidate for further investigation into inducers of late-stage antimicrobial-refractory Lyme arthritis than the OspA_163–175_ epitope. Although OspA is considered a leading candidate that induces arthritis in humans, other *B. burgdorferi* antigens, as well as intrinsic host factors and host HLA-DR alleles, may also play a role in the induction of arthritis. Such considerations provide a tremendous impetus for the elucidation of mechanisms that result in severe destructive Lyme arthritis, especially when considering that OspA or other antigens are prominent Lyme borreliosis vaccine candidates.

It is known that two clinical entities of Lyme borreliosis articular involvement occur in humans. It is critical to make the distinction between synovitis and severe destructive Lyme arthritis. This review will focus on the utility of the hamster and murine models in experimental investigations of severe destructive Lyme arthritis. Each animal model of an articular condition analogous to that which afflicts humans in early Lyme borreliosis will be discussed. This will be followed by presentation of experimental models of severe destructive Lyme arthritis. Benefits and applications of these models will subsequently be expounded upon.

## 2. Hamster Models of Synovitis and Severe Destructive Lyme Arthritis

### 2.1. Synovitis

When immunocompetent adult London School of Hygiene (LSH) hamsters were challenged in the footpad with 10^6^ live *B. burgdorferi sensu stricto* isolate 297, a slight degree of tibiotarsal joint swelling was observed between 10 and 14 days after inoculation [18]. In addition, histopathological changes were demonstrated in these animals. Synovial hyperplasia with focal areas of ulceration and overlying adherent fibrin was present at one week of infection. The majority of immune cells responsive to this inoculum were neutrophilic in nature, with this infiltrate involving the synovial and periarticular structures, including the fibrous capsule, periosteum, ligaments, tendons, and tendon sheaths. Numerous spirochetes were also observed through the use of silver impregnation staining techniques.

Such histological evidence of synovitis persisted for approximately three weeks, after which both macroscopic edema and histopathological involvement waned. Following a 15-week observation of infected hamsters, histopathological examination noted that subsynovial and periarticular soft tissues contained increased fibroblasts and collagen, persistent small hypertrophic synovial villi were still evident, and spirochetes were no longer seen. No destructive bone erosion was reported. No edematous or histopathological findings were seen in hamsters challenged in the footpad with growth medium.

This model is used to investigate the innate immune response to infection with *B. burgdorferi*. Since humans develop arthritis weeks to months after infection, the hamster model of synovitis is valuable for understanding the early immune events that drive the neutrophilic response to infection, induction of cytokines, and early antigenic processing that are responsible for the later induction of adoptive immunity. Most investigations using mice, hamsters, and other animals have focused on this early stage of development of arthritis. Therefore, there is considerable debate over whether T lymphocytes are responsible for induction of arthritis. Other animal models (see the following) that focus on nonearly events strongly support a role for T lymphocytes in the induction and resolution of arthritis. Most important, the early innate immune responses responsible for the induction of synovitis are directly connected to the T lymphocyte responses that drive the more severe pathology of severe destructive Lyme arthritis.

### 2.2. Irradiation Model of Severe Destructive Lyme Arthritis

Adult LSH hamsters that were subjected to 550 rads of gamma radiation manifested a tibiotarsal joint swelling upon footpad challenge with 10^6^ live *B. burgdorferi* 297 [18]. Edema peaked approximately ten days after challenge and was found to be significantly elevated over that of nonirradiated infected hamsters throughout the first five weeks of observation. Furthermore, behavioral changes were observed in the irradiated infected animals. These hamsters were increasingly irritable and vicious, maneuvering about their cages very slowly on their abdomens, dragging their hind legs. Analogous behavior was not observed in nonirradiated infected hamsters.

Histopathological examination of infected irradiated hamsters demonstrated a more severe and fulminate articular involvement. Greater synovial hyperplasia and hypertrophy were observed to the point that pannus formations bridging synovial spaces were noted. Neutrophilic infiltrate resulted in erosion of articular cartilage and superficial destruction of underlying bone. Increases in collagen formation and numbers of fibroblasts were shown also to result in fibrosing ankylosis, loss of alignment of the articular surfaces, and overall deformation of the joints [19]. This animal model is used to evaluate the adoptive immune responses that protect against infection. Cells or sera from immunized or naturally infected animals are transferred to irradiated hamsters and then challenged with *B. burgdorferi* to determine their role in protection. In addition, cells and cytokines participating in preventing or augmenting the arthritis can be determined.

### 2.3. Immunocompetent Model of Severe Destructive Lyme Arthritis

The relevance of experimental hamsters was further established with the advent of the immunocompetent hamster model of severe destructive Lyme arthritis. When Lim et al. [20] vaccinated adult LSH hamsters with formalin-inactivated *B. bissettii *(formerly *B. burgdorferi sensu stricto* isolate C-1-11) in aluminum hydroxide adjuvant (alum), gross tibiotarsal joint edema was observed upon footpad challenge with heterologous isolates of *B. burgdorferi* or with *B. bissettii* prior to the induction of protective immunity. Edema measurements demonstrated statistically significant increases in tibiotarsal swelling over those observed in nonvaccinated hamsters challenged with multiple isolates of *B. burgdorferi*. It was also shown that arthritis could be induced using live spirochetes, heat-inactivated spirochetes, or antimicrobial-treated organisms mixed in alum. Only viable organisms, however, were required upon challenge to elicit the arthritis.

Analogous to differences observed macroscopically, histopathological examination revealed varying degrees of arthritis severity in vaccinated and nonvaccinated hamsters following challenge with *B. burgdorferi*. Erosion of articular cartilage, focal destruction of underlying bone, and chronic hyperplasia and hypertrophy characterized by pannus formation were observed in the synovia of hamsters challenged with *B. bissettii* following a five-week vaccination with *B. bissettii* in adjuvant. These animals also manifested a cellular infiltrate of neutrophils, macrophages, lymphocytes, mast cells, and plasma cells in the subsynovial and periarticular tissues. In contrast, nonvaccinated hamsters challenged with *B. bissettii* displayed an acute synovitis characterized by a cellular inflammatory infiltrate devoid of pannus formation or bone erosion. Severe destructive Lyme arthritis can also be induced using other isolates of *B. burgdorferi* mixed in alum and then infected with a homologous spirochete before borreliacidal antibody develops or with a heterologous isolate when isolate-specific borreliacidal antibody is present.

This model mimics how humans develop Lyme arthritis. Humans develop Lyme arthritis many weeks after they are infected/vaccinated by spirochetes that entered the host by the bite of an infected tick. Persistence of the spirochete or its antigens, such as OspA, in the nontreated individual would initiate a series of immunological events in the host that finally induce arthritis. By vaccinating hamsters, the immunological events responsible for the induction of arthritis are compressed from weeks into days. A major concern with the hamster model of severe destructive Lyme arthritis is the lack of immunological reagents that can be utilized to decipher the immune components responsible for the induction of arthritis.

#### 2.3.1. Utilization of Hamster Models of Severe Destructive Lyme Arthritis to Assess Protective Immunity

A feature common to both the irradiated and immunocompetent LSH hamster models of severe destructive Lyme arthritis is their utility in characterizing and monitoring protective immunity. Schmitz et al. [21] reported that heat-inactivated *B. burgdorferi*-immune sera were able to inactivate inocula of live *B. burgdorferi* in the presence of complement in an *in vitro* assay. These specific killing antibodies were thus termed borreliacidal antibodies. To determine the protective capability of these borreliacidal antibodies, irradiated hamsters received sera through adoptive transfer and were subsequently challenged with *B. burgdorferi*. Irradiated hamsters receiving sera with low titer of borreliacidal antibody developed severe destructive Lyme arthritis while hamsters receiving sera containing significant borreliacidal activity were protected from *B. burgdorferi* infection, as well as the evocation of severe destructive Lyme arthritis. In similar fashion, Callister et al. [22] used the irradiated hamster model of severe destructive Lyme arthritis to prove that humans generate a borreliacidal response upon infection with *B. burgdorferi*. Adoptive transfer of sera from humans with clinically diagnosed Lyme borreliosis (significant borreliacidal antibody response detected *in vitro*) protected irradiated hamsters from *B. burgdorferi* infection and subsequent progression to severe destructive Lyme arthritis, while transfer of sera from healthy individuals (no borreliacidal activity detected *in vitro*) failed to protect hamsters from induction of severe destructive Lyme arthritis. Taken together, these data suggest that *in vitro* borreliacidal activity correlates with protection events *in vivo*.

Induction of these borreliacidal antibodies establishes the effectiveness of Lyme borreliosis vaccine preparations [23]. When Jobe et al. [24] actively immunized irradiated hamsters with a commercial canine whole cell Lyme borreliosis vaccine, gross tibiotarsal joint swelling, and severe destructive Lyme arthritis were induced in these animals when challenged three weeks after vaccination with live *B. burgdorferi*. Conversely, animals challenged five weeks after vaccination were protected from such manifestations. Similarly, when Lim et al. [20] vaccinated immunocompetent hamsters with formalin-inactivated *B. bissettii* in aluminum hydroxide, severe destructive Lyme arthritis was induced in these animals when challenged with a homologous isolate of *B. burgdorferi* at intervals of less than seven weeks after immunization. By contrast, hamsters challenged with *B. bissettii* seven and nine weeks after vaccination failed to manifest severe destructive Lyme arthritis. Subsequent experiments [25] revealed that hamsters vaccinated with formalin-inactivated *B. burgdorferi* isolate 50772 in aluminum hydroxide were protected against a subsequent homologous challenge at time points ranging from 5 to 18 weeks after vaccination. Severe destructive Lyme arthritis was induced in vaccinated hamsters that were challenged at intervals both earlier (10 days, 3 weeks) and later (24 weeks) than those which afforded protection. These data exemplify how irradiated and immunocompetent hamsters have been used as an *in vivo* correlate to a common concern pertaining to Lyme disease vaccine preparations that was first observed *in vitro* [24, 26], namely, relatively short duration of protective borreliacidal antibody production. Therefore, these models can be utilized to validate the duration of protection induced in individuals receiving any future Lyme borreliosis vaccine. They may also establish the number of vaccinations required to establish long-term protection.

Irradiated and immunocompetent hamster models of severe destructive Lyme arthritis have also been implemented to investigate an additional pitfall with regard to Lyme borreliosis vaccine preparations. Seroprotectivity is an index of the ability of immune sera generated in one mammalian host to protect, via adoptive transfer, a naïve recipient animal against subsequent challenge. As an example, irradiated hamsters that were passively immunized with sera generated by the infection of hamsters with *B. burgdorferi* 297 were protected from the induction of severe destructive Lyme arthritis upon challenge with *B. burgdorferi sensu stricto* isolate B31. Conversely, passive administration of anti-*B. burgdorferi* 297 sera failed to protect irradiated hamsters from the evocation of severe destructive Lyme arthritis upon challenge with *B. bissettii* [27]. On the basis of these and other data [28], Lovrich et al. established six seroprotective groups of *B. burgdorferi sensu lato* isolates, with inherent differences in seroprotectivity being partially reflective of the worldwide distribution of *B. burgdorferi sensu lato* isolates. Immunocompetent hamsters were utilized in a similar fashion to classify isolate-specific rOspA preparations into analogous seroprotective groups [29], signaling an additional concern pertaining to any new federally licensed Lyme borreliosis vaccine preparations. These models can be easily utilized to assess the degree of cross-protection and development of a universal vaccine.

#### 2.3.2. Utilization of Hamster Models of Severe Destructive Lyme Arthritis to Assess the Role of Cell Populations in Induction of Severe Destructive Lyme Arthritis

The findings of Lim et al. [20] were important for an additional reason. This published model of severe destructive Lyme arthritis did not require the utilization of gamma radiation. This allowed for elucidation of roles that certain cell populations play in the induction of severe destructive Lyme arthritis in the context of an immunocompetent mammalian host. Du Chateau et al. [30] reported that macrophages play a direct effector role in the induction of severe destructive Lyme arthritis. Macrophages that were obtained from vaccinated or nonvaccinated LSH hamsters, exposed *in vitro* to formalin-inactivated *B. burgdorferi*, and subsequently infused into footpads of naïve recipients induced severe destructive Lyme arthritis upon challenge with *B. burgdorferi*. Both the severity and onset of the arthritis were dependent on the number of macrophages infused into the recipient footpads. In addition, macrophages not exposed to *B. burgdorferi in vitro* failed to induce severe destructive Lyme arthritis upon *B. burgdorferi* challenge. Roles for T lymphocytes and subset populations thereof, either alone [31, 32] or in synergy with macrophages [33, 34], have been proposed in the pathology of severe destructive Lyme arthritis. However, the value of the hamster model of severe destructive Lyme arthritis in further studies of the immunoregulation of severe destructive Lyme arthritis has been constrained due to the unavailability of many hamster-specific molecular and immunological reagents.

#### 2.3.3. Immunocompetent Model of Severe Destructive Lyme Arthritis for Assessment of Lyme Borreliosis Vaccine Preparations

An extremely practical and relatively straightforward application of the immunocompetent LSH hamster model of severe destructive Lyme arthritis involves safety assessment of Lyme borreliosis vaccine preparations. Speculation that the severe destructive Lyme arthritis was elicited in hamsters only because a whole cell component of *B. burgdorferi* was used as the immunogen was refuted by Croke et al. [35]. Vaccination of immunocompetent LSH hamsters with 30 *μ*g of recombinant (r) OspA in alum primed these hosts for the induction of gross macroscopic edema and corresponding severe destructive Lyme arthritis upon footpad challenge with both heterologous and homologous isolates of *B. burgdorferi*. By contrast, hamsters that were vaccinated with 30 *μ*g of rOspA in alum and not subsequently challenged with *B. burgdorferi* manifested no tibiotarsal joint edema. Intact joints and normal capsular and pericapsular soft tissue were noted upon histopathological examination. The rOspA vaccine preparation that was shown to prime hamsters for induction of deleterious effects is analogous to commercial vaccine preparations [36, 37] that were licensed by the United States Food and Drug Administration for use in humans. These data further confirm the importance of the immunocompetent hamster model of severe destructive Lyme arthritis for the determination of possible deleterious effects that commercial Lyme borreliosis vaccines may evoke in mammalian hosts. As additional support, Croke et al. [35] also demonstrated that a commercial canine rOspA vaccine prepared in alum primed hamsters for the induction of severe destructive Lyme arthritis upon challenge with *B. burgdorferi*. Obviously, this model should be utilized to evaluate vaccine preparations for adverse effects before clinical trials begin.

## 3. Murine Models of Synovitis and Severe Destructive Lyme Arthritis

### 3.1. Synovitis

In early investigations, it was determined that the strain of mouse was important for the degree of Lyme borreliosis articular involvement potentiated [38–40], which, in turn, was correlated to the MHC haplotype of the mouse. C57BL/6 mice (H2*^b^*) were resistant to the induction of arthritic manifestations in spite of infection with a large inocula of *B. burgdorferi*, while BALB/c mice (H2*^d^*) developed a mild articular condition. In contrast, strains of H2*^k^* haplotype mice, including AKR and C3H, manifest a Lyme arthropathy upon challenge with *B. burgdorferi*. The edema and synovitis observed in these infected mice closely resembled those of adult immunocompetent LSH hamsters challenged with *B. burgdorferi* [41, 42]. Endotoxin susceptibility differences inherent to the C3H mouse did not contribute to potentially varying degrees of arthropathy, as endotoxin-resistant C3H/HeJ mice manifest equivalent synovitis to that of endotoxin-susceptible C3H/HeN mice [43].

Although the C3H mouse is the most frequently utilized animal model to investigate the immune mechanisms of Lyme arthritis, the model has limitations. Mice develop synovitis within days of infection. Humans do not develop synovitis within days after infection with *B. burgdorferi*. In addition, the arthritis in mice is mild and is not sustained. Moreover, most C3H mice resolve the arthritis 3 to 6 weeks after infection. Rapid resolution of arthritis also does not occur in humans infected with *B. burgdorferi*. The model is utilized to determine the early innate immune events that occur when spirochetes enter the host after being bitten by a *Borrelia*-infected tick. Therefore, this model may not mimic the immunological events that drive the induction of severe arthritis in humans.

### 3.2. Murine Models of Severe Destructive Lyme Arthritis

#### 3.2.1. Immunodeficient Mouse Models

Multiple studies [44–46] have reported that challenge of SCID mice with *B. burgdorferi* induces a severe destructive Lyme arthritis. When Schaible et al. [44] challenged CB.17 SCID mice subcutaneously in the tail with *B. burgdorferi sensu stricto* isolate ZS7 or ZQ1, inoculum-dependent clinical signs of arthritis were first observed between days 7 and 20 after infection. Mice developed redness and swelling of tibiotarsal joints and were observed to have difficulty walking. Such manifestations persisted throughout the entire observation period of 87 days. Histopathological examination characterized arthritic involvement as synovial hyperplasia, infiltration of mononuclear leukocytes into inflamed synovium, and the formation of pannus with joint erosion and cartilage destruction. In contrast, normal CB.17 mice did not develop any histopathological alterations following challenge with *B. burgdorferi*. A subsequent study by Schaible et al. [45] extended observations of CB.17 SCID mice to 195 days after challenge with *B. burgdorferi* ZS7. Macroscopic manifestations of arthritis failed to resolve even after this interval and severe destructive arthritic involvement progressed from tibiotarsal joints to the metatarsal and ulnocarpal joints. Furthermore, histopathological changes were noted in the ligaments, tendons, fascia, and skeletal muscle.

Barthold et al. [46] also compared the progression of Lyme arthritis in C3H/He SCID mice to that observed in C3H/He mice. While the maximum severity and distribution of arthritis were observed at relatively early intervals after intradermal infection with *B. burgdorferi sensu stricto* isolate N40, arthritis severity progressively worsened in C3H/He SCID mice throughout a 60-day observation period. Histopathological analysis of infected C3H/He SCID mice 60 days after infection revealed very severe joint disease involving nearly all tibiotarsi, knees, elbows, and carpi examined. Intensely proliferating synoviocytes caused effacement of joints, tendons, and bursae. Obliteration of the joint cavity and losses of cartilage and bone were noted. Such characterization of histopathology is consistent with the clinical entity of severe destructive Lyme arthritis. By contrast, at a peak interval of articular involvement in C3H/He mice (between 14 and 30 days after *B. burgdorferi* challenge), tibiotarsal joints displayed moderate synovial proliferation, leukocytic infiltration, and exudation into the joint lumen. The immunodeficient mouse model may not represent the actual events that occur in humans. However, the model is important for understanding the components of the immune response involved in the induction of arthritis. Without this model, the complexity of the immune response participating in the development of synovitis would be extremely difficult to decipher.

#### 3.2.2. Irradiated Mouse Model

Irradiation of mice followed by infection with *B. burgdorferi* can also induce severe destructive Lyme arthritis. Low-passage (*P* < 5) *B. burgdorferi* 297 was cultivated at 32°C in Barbour-Stoenner-Kelly (BSK) medium [47] containing screened lots of bovine serum albumin [48]. Two groups of three C3H/HeJ mice each were subjected to 550 rads of gamma radiation with a ^60^Co irradiator (Picker Corporation, Cleveland, OH). Mice were then immediately anesthetized with methoxyflurane and footpads of one group of mice were infused with 0.2 mL of a 5 × 10^6^/mL suspension of *B. burgdorferi* 297. The second group of irradiated mice was injected in the footpad with 0.2 mL of sterile BSK. A group of three C3H/HeJ mice, not subject to gamma radiation, was challenged in the footpad with 10^6^ *B. burgdorferi* 297 delivered in a 0.2 mL volume.

**Figure 1:**
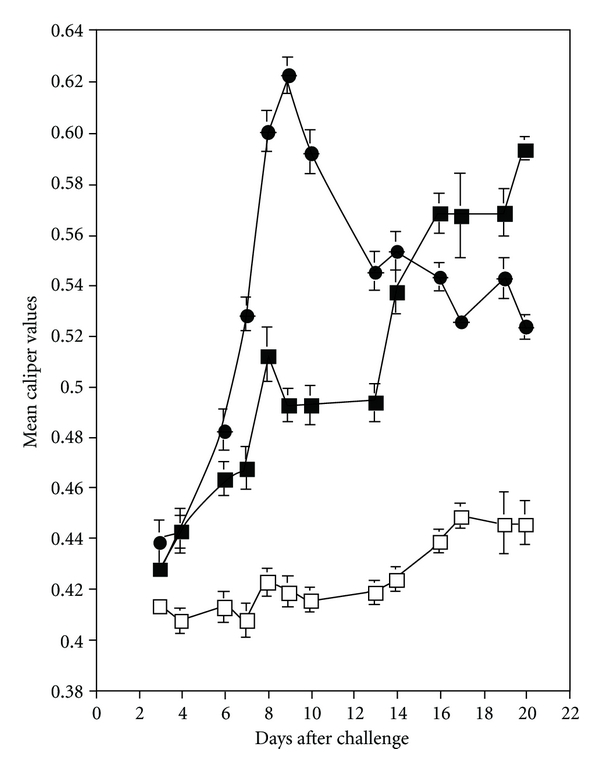
Swelling of the tibiotarsal joint detected in irradiated C3H/HeJ mice injected with *B. burgdorferi sensu stricto* isolate 297 (■) or BSK medium (□) and in nonirradiated C3H/HeJ mice infused with *B. burgdorferi* 297 (●).

Inflammatory responses were assessed at the macroscopic level for 20 days by carefully measuring the width and thickness of each tibiotarsal joint with a dial-type vernier caliper (Fisher Scientific, Pittsburgh, PA), combining these values, and determining mean dimensions within a given group. Tibiotarsal joint edema peaked nine days after challenge of nonirradiated mice with *B. burgdorferi* and began to wane thereafter (Figure 1). By contrast, onset of edema was slightly delayed in irradiated mice that were challenged with *B. burgdorferi*, with swelling beginning to peak 20 days after challenge (Figure 1). Both groups of mice challenged with *B. burgdorferi* manifested peak edema that was relatively equal to each other and exceeded that observed in mice injected with BSK medium.

**Figure 2:**
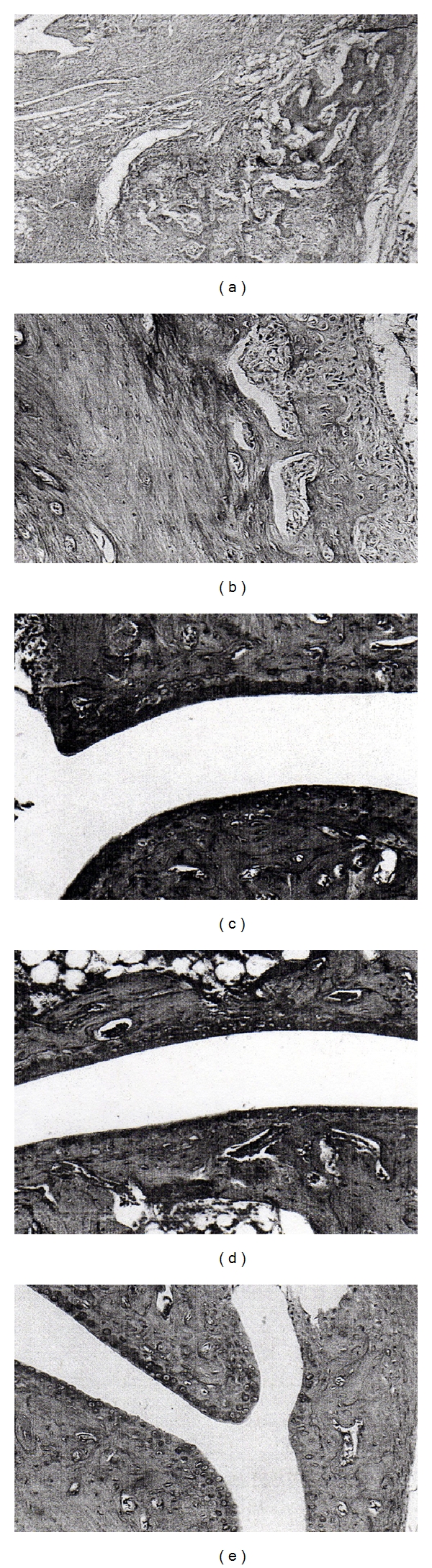
Histopathological responses detected in irradiated ((a) and (b)) and nonirradiated ((c) and (d)) C3H/HeJ mice injected with *B. burgdorferi sensu stricto *isolate 297. Irradiated control mice (e) were injected with BSK medium. All specimens were acquired 21 days after challenge, with the exception of panel (c), day 14. Panels (a) and (b) were viewed with a 4x or 10x objective lens, respectively.

After 21 days of observation, mice were euthanized by CO_2_ asphyxiation and hind legs of all mice were amputated at the midfemur and fixed in 10% neutral buffered zinc formalin (Fisher). While peak edema measurements between infected irradiated and nonirradiated mice were similar, the degree of arthropathy was distinctly different between the two treatment groups. A severe destructive Lyme arthritis was induced in the hind legs of irradiated mice challenged with *B. burgdorferi*. This was characterized, in part, by a dense infiltration of inflammatory cells which compacted synovial spaces and by widespread erosion of articular cartilage and destruction of small bones (Figures 2(a) and 2(b)). This was in stark contrast to the histopathological response of hind legs of nonirradiated mice infected with *B. burgdorferi* which demonstrated a mild synovial hyperplasia and joint spaces free of inflammatory cells (Figure 2(d)). Examination of histopathology from infected nonirradiated mice at an interval (day 14) closer to that at which peak macroscopic edema was observed revealed a similar mild Lyme synovitis (Figure 2(c)). Mice injected with BSK medium failed to present any articular involvement (Figure 2(e)). Although this model is infrequently utilized, it is valuable to assess the role of immune components in protection and the development of severe destructive Lyme arthritis.

#### 3.2.3. Interferon-Gamma- (IFN-*γ*-) Deficient Mouse Model

Classically, a T helper lymphocyte type 1 (Th1) response picture has been painted to explain the induction of Lyme arthritis. Humans with clinically diagnosed Lyme borreliosis produce increased serum levels of the Th1 cytokine IFN-*γ* and decreased quantities of interleukin-(IL-) 4 when compared to healthy individuals [54]. Yang et al. [55] noted that mice susceptible to synovitis following *B. burgdorferi* infection produced increased serum levels of IgG_2a_, an immunoglobulin isotype associated with a Th1 response. Independent groups [56, 57] reported that C3H mice produced IFN-*γ* following *B. burgdorferi* infection, while BALB/c mice, with an inherent variable susceptibility to synovitis, produced IL-4 following challenge with *B. burgdorferi*. Furthermore, treatment of BALB/c mice with neutralizing antibody to murine IL-4 facilitated the development of synovitis upon challenge with *B. burgdorferi*, whereas treatment of C3H mice with rIL-4 or neutralizing antibody to murine IFN-*γ* reduced the severity of synovitis [56–58].

On the other hand, studies have presented instances in which decreases in IFN-*γ* production or an inherent deficiency of IFN-*γ* had no impact on arthritis severity. Blockage of the B7-CD28 interaction in BALB/c mice resulted in an increase of IFN-*γ* production with no concomitant change in synovitis [59]. Depletion of natural killer cells in C3H/HeJ mice resulted in a loss of early IFN-*γ* production with no alteration of synovitis [60]. Direct evidence not supporting a role for IFN-*γ* in synovitis induction was presented by Brown and Reiner [61]. Upon histopathological examination of tibiotarsal joints, IFN-*γ*-deficient mice that were challenged with *B. burgdorferi* were shown to manifest equivalent synovitis to that observed in infected wild-type controls.

An *in vitro* neutralization model [50] supplied additional evidence in support of the hypothesis that IFN-*γ* does not play a significant role in the induction of articular manifestations of Lyme borreliosis. C3H/HeJ mice were immunized with formalin-inactivated *B. burgdorferi* in alum. Inguinal lymph node cells were cocultured with *B. burgdorferi* and macrophages and were treated *in vitro* with either rIFN-*γ* or neutralizing antibody to IFN-*γ*. Cultures were incubated for 24 hours at which time contents were infused into footpads of recipient C3H/HeJ mice. While three-week edema measurements did not vary significantly between recipients of immune lymph node cells treated with rIFN-*γ* or anti-IFN-*γ*, histopathological examination of hind legs infused with cells treated *in vitro* with anti-IFN-*γ* manifested severe destructive Lyme arthritis [50]. In contrast, both recipients of immune lymph node cells treated *in vitro* with rIFN-*γ* and recipients of nontreated cultures of cells obtained from vaccinated or nonvaccinated C3H/HeJ mice demonstrated synovitis.

A clearer picture of the role of IFN-*γ* on the development of severe destructive Lyme arthritis was shown by Christopherson et al. [51]. When C57BL/6 IFN-*γ*-deficient mice were vaccinated with Formalin-inactivated *B. burgdorferi* in alum, gross tibiotarsal edema and a corresponding severe destructive arthritis were induced upon footpad challenge with a heterologous isolate of *B. burgdorferi* [51]. Challenge of nonvaccinated mice produced only a mild synovitis and tibiotarsal joint measurements that were significantly less than those observed in vaccinated mice but slightly greater than those measured in IFN-*γ*-deficient mice inoculated with BSK medium. These data corroborated those of Brown and Reiner [61] that IFN-*γ* is not required for the development of arthropathy in an infection model of murine Lyme borreliosis.

The findings of Christopherson et al. [51] also suggested that inflammatory pathways other than a traditional Th1, IFN-*γ*-mediated response may stimulate the development of severe destructive Lyme arthritis following infection with *B. burgdorferi*. Using the IFN-*γ*-deficient murine model, Burchill et al. [52] demonstrated a significant role for the proinflammatory cytokine IL-17 in the induction of severe destructive Lyme arthritis. When these mice were vaccinated and challenged three weeks later with a heterologous bacterial strain in the footpad, they developed inflammatory changes analogous to those observed by Christopherson et al. [51]. However, administration of antibodies to either IL-17 or the IL-17 receptor on the day of infection and daily thereafter for 11 days reduced the severity of hind paw edema and abrogated the development of severe destructive Lyme arthritis [52].

These findings were extended by Nardelli et al. [53] who showed that administration of anti-IL-17 to *Borrelia*-vaccinated and -infected IFN-*γ*-deficient C57BL/6 mice also correlated with an inordinate increase in the numbers of CD4^+^CD25^+^ T lymphocytes in local lymph nodes. Concomitant depletion of IL-17 and CD4^+^CD25^+^ T lymphocytes from these *Borrelia*-vaccinated and -infected mice resulted in massive edema of the hind paws, extensive erosion of cartilage, bone, and synovial tissue of the tibiotarsal joints, and pannus formation into the joint space, indicating that these cells played a significant role in the protection from *Borrelia*-induced arthritis mediated by neutralization of IL-17 [53]. In support of this, depletion of only CD4^+^CD25^+^ T lymphocytes from *Borrelia*-vaccinated and -infected mice resulted in inflammatory changes similar to those of *Borrelia*-vaccinated and -infected control mice [62]. Significantly, adoptive transfer of CD4^+^CD25^+^ T lymphocytes obtained from anti-IL-17-treated *Borrelia*-vaccinated and -infected mice wholly prevented histopathological changes of the hind paws [63]. This suggested that those CD4^+^CD25^+^ T lymphocytes which developed in the absence of IL-17-mediated inflammation conferred protection against the induction of arthritis. Therefore, the development of arthritis in this murine model may be attributed to the combined effects of IL-17 production and a correspondingly low presence of CD4^+^CD25^+^ T lymphocytes. These findings were obtained using IFN-*γ*-deficient mice, which allows investigators to determine the interaction among cytokines that directly or indirectly are affected by IFN-*γ*.

Collectively, these findings not only provided potentially valuable insights into the pathogenesis of severe destructive Lyme arthritis, but also have played a significant role in elucidating immunological pathways beyond the realm of Lyme borreliosis. In support of this statement, Codolo et al. [64] showed that synovial T lymphocytes isolated from humans with Lyme arthritis secrete IL-17 upon stimulation with neutrophil activating protein A of *B. burgdorferi*. In addition, Shen et al. [65] observed the presence of Th17 cells in the synovial fluid of humans with Lyme arthritis; however, their direct role in mediating the induction or propagation of Lyme arthritis was not determined. Moreover, these early investigations of the role of IL-17 in the development of murine Lyme arthritis were used to support the seminal immunological findings of Bettelli et al. [66] that Th17 cells and CD4^+^CD25^+^Foxp3^+^ regulatory T lymphocytes develop in a reciprocal manner from a common precursor cell, and that this development is governed by the presence or absence of an inflammatory environment.

#### 3.2.4. Immunocompetent Mouse Model of Severe Destructive Lyme Arthritis

Although use of IFN-*γ*-deficient mice to study the role of cytokines in the development of severe destructive Lyme arthritis is valuable, severe arthritis can also be induced in C57BL/6 mice. Mice are vaccinated in the inguinal region with washed spirochetes mixed with 1% alum. Approximately three weeks after vaccination, mice are challenged with viable *B. burgdorferi*. This animal model was utilized to determine the role of IL-35 on the production of IL-17 [49].

IL-35 is a major cytokine produced by CD4^+^CD25^+^Foxp3^+^ regulatory T lymphocytes. Based on previous findings that administration of anti-IL-17 prevented arthritis in *Borrelia*-vaccinated and -infected mice and induced production of CD4^+^CD25^+^ T lymphocytes with immunoregulatory function, we hypothesized that IL-35 would decrease the severity of arthritis by inhibiting the production of IL-17 in *Borrelia*-vaccinated and -infected mice. When IL-35 was administered to *Borrelia*-vaccinated and -infected mice, IL-35 enhanced arthritis. This suggests that IL-35 does not play a major role in preventing the induction of arthritis. Additional investigations are needed to determine which cytokines are responsible for the induction and resolution of arthritis. These studies are important because they may lead to a therapeutic agent that may decrease the effects of arthritis. The mouse model of severe destructive Lyme arthritis yields reproducible data and hopefully the results can be applied to humans with Lyme arthritis.

## 4. A Unifying Hypothesis

Using the data obtained through the investigation of hamster and mouse models, as well as through analysis of human tissues *ex vivo*, a unifying hypothesis can be developed to elucidate the mechanisms by which severe destructive Lyme arthritis is induced. Central to the development of this chronic manifestation are (1) the stimulation of innate immune effectors following infection with *B. burgdorferi*, with subsequent stimulation of adaptive immune mechanisms; (2) survival and persistence of the spirochete in the face of a robust innate immune response and increasing (but eventually waning) borreliacidal antibody titers, and (3) recognition of *B. burgdorferi* or its proteins by primed T lymphocytes, with ensuing tissue-destructive effects occurring in the joint.

Upon bloodmeal acquisition by infected *Ixodes* spp., host macrophages, dendritic cells, and/or their monocyte precursors rapidly recognize lipoproteins of *B. burgdorferi* via Toll-like receptor 2. Processing of the spirochete by these cells stimulates the secretion of various cytokines, such as tumor-necrosis factor-*α*, interleukin-1*β*, and interleukin-6, [64, 67], which serve to recruit neutrophils and amplify the early inflammatory events manifesting as synovitis. Some spirochetes survive this initial innate immune defense, as well as the borreliacidal antibody response triggered by innate effectors. *B. burgdorferi* disseminates to the articular tissues, where the spirochetes or their proteins may be recognized weeks, months, or years later to activate a new wave of innate immunity as well as previously primed adaptive immune cells. Innate immune cells in the synovial tissues and/or fluid may again secrete an array of cytokines, including transforming growth factor-*β*, IL-6, IL-12 (among others) that polarize the T lymphocyte response toward inflammatory subsets such as Th1 and Th17 cells. In turn, the immune response would be directed away from T lymphocyte subsets, such as Th2 and regulatory T lymphocytes, which antagonize these inflammatory populations. Both Th1 and Th17 responses have been postulated to play a role in later-stage Lyme arthritis [68], which is supported by observation of these cells in the synovial fluid of humans with Lyme arthritis [65], implication of their prototypical cytokines in disease [52, 69], and recognition that these cytokines induce severe destruction in other models of arthritis [70, 71]. These inflammatory responses may persist until the antigenic stimulus is eventually cleared, at which point regulatory T lymphocytes may mediate suppression of inflammation and tissue remodeling may occur.

## 5. Conclusions

Selection of the proper animal model is paramount in relevant investigations of articular involvement of Lyme disease. Delivery of *B. burgdorferi* to immunocompetent LSH hamsters and C3H mice elicits a mild and transient synovitis that mimics pathology observed in individuals in early Lyme borreliosis. However, in order to investigate the chronic destructive arthropathy with which a certain subpopulation of individuals in late Lyme borreliosis may be afflicted, one must choose either the immunocompetent hamster model of severe destructive Lyme arthritis or various murine models of severe destructive Lyme arthritis that manifest a degree of immunodeficiency (Table 1).

While murine models, with their replete armamentarium of immunological reagents, appear very promising in the future study of severe destructive Lyme arthritis, we do not intend to eulogize the hamster model of severe destructive Lyme arthritis. The immunocompetent hamster model of severe destructive Lyme arthritis can be a very advantageous system for safety assessment of Lyme borreliosis vaccine preparations (should redevelopment efforts ever come to fruition [72, 73]) due to the fact that macroscopic observation of footpad edema correlates well with the severity of histopathology (Table 2).

Murine systems of severe destructive Lyme arthritis will provide a tremendous advantage in the future elucidation of immunological mechanisms that result in severe destructive Lyme arthritis. In particular, the advent of a system in which immune cells from an immunocompetent host are programmed *in vitro* to elicit severe destructive Lyme arthritis upon subsequent transfer into a recipient immunocompetent host presents great potential in the ultimate elucidation of the mechanisms of severe destructive Lyme arthritis. In addition, this system may also allow for simultaneous assessment of Lyme borreliosis vaccine candidates (following selection of protective epitopes [74]) for production of protective borreliacidal antibodies *in vitro* [50, 75–78] as well as the deleterious capability of priming recipients for induction of severe destructive Lyme arthritis [50]. Taken together, these investigations may contribute to the production of a safe and efficacious vaccine to prevent *B. burgdorferi* infection.

## Figures and Tables

**Table 1:** Hamster and murine models important for evaluation of the pathogenesis of severe destructive Lyme arthritis.

Model	*B. burgdorferi *strains	Timing of arthritogen delivery	Result	Comments	Reference(s)
Immunocompetent hamster (challenge)	Numerous^a^	Can vary	Synovitis	Studying innate responses	[18]
Immunocompetent mouse (challenge)	Numerous^a^	Can vary	Synovitis	Studying innate responses	[38–42]
Irradiated hamster (challenge)	Numerous^a^	Following 550 rads gamma radiation	SDLA^b^	Insight into cellular components responsible for SDLA	[18, 19]
Irradiated mouse (challenge)	297	Following 550 rads gamma radiation	SDLA	Insight into cellular components responsible for SDLA	Stated in this publication
SCID mouse (challenge)	Numerous	Can vary	SDLA	Insight into cellular components responsible for SDLA	[44–46]
Immunocompetent hamster (vaccination/challenge)	Numerous^c^	Challenge prior to development of significant borreliacidal antibody titer (homologous challenge)^d^; timing of heterologous strain challenge can vary	SDLA	Insight into adoptive cellular components responsible for SDLA in an immunocompetent host	[20, 30–34]
Immunocompetent mouse (vaccination/challenge)	297	Challenge following 21 days of vaccination	SDLA	Elucidating adoptive immunological pathways of SDLA in an immunocompetent host	[49]
*In vitro* murine IFN-*γ* neutralization	297	Lymph node cells pulsed *in vitro *	SDLA	Addition of IFN-*γ* to lymph node cells prior to *in vitro B*. *burgdorferi *pulse; footpad challenge of naïve mice following cell culture transfer; Assessment of arthritogenic potential of *B. burgdorferi* immunogens	[50]
IFN-*γ*-deficient mouse (vaccination/challenge)	297	Challenge following 21 days of vaccination	SDLA	Elucidating immunological pathways of SDLA	[51–53]

^a^Referenced study(ies) focused on *B. burgdorferi sensu stricto* isolate 297.

^b^Severe destructive Lyme arthritis.

^c^Referenced study(ies) focused on *B. bissettii* vaccination with subsequent *B. burgdorferi sensu stricto* isolate 297 challenge.

^d^At least six seroprotective groups have been characterized ([27–29]).

**Table 2:** Hamster models important for evaluation of protective immunity versus endpoint of severe destructive Lyme arthritis.

Model	*B. burgdorferi *strains	Timing of immunogen delivery	Endpoint	Comments	Reference(s)
Irradiated hamster (challenge)	Numerous^a^	Following 550 rads gamma radiation	SDLA^b^	Adoptive transfer (protection) experiments	[18, 21, 22]
Irradiated hamster (vaccination/challenge)	Numerous^a^	Challenge prior to development of significant borreliacidal antibody titer (homologous challenge)^c^; timing of heterologous strain challenge can vary	SDLA	Assessment of vaccine candidates	[24]
Immunocompetent hamster (vaccination/challenge)	Numerous^d^	Challenge prior to development of significant borreliacidal antibody titer (homologous challenge)^c^; timing of heterologous strain challenge can vary	SDLA	Characterizing duration of protective immunity of vaccine candidates; Assessing Lyme borreliosis vaccine candidates for adverse events	[20, 25] [29, 35]

^a^Referenced study(ies) focused on *B. burgdorferi sensu stricto* isolate 297.

^b^Severe destructive Lyme arthritis.

^c^At least six seroprotective groups have been characterized ([27–29]).

^d^Referenced study(ies) focused on *B. bissettii* vaccination with subsequent *B. burgdorferi sensu stricto* isolate 297 challenge.
